# The Transactions of the American Medical Association, Held at Chicago, June 2, 1863

**Published:** 1864-05

**Authors:** 


### REVIEW.

*The Transactions of the American Medical Association, held at Chicago, June* 2, 1863.

We have received this volume too late to speak in detail of the interesting papers it contains. We hope hereafter to be able to do so, and for the present simply announce its appearance and give the table of contents.

*Contents.*—Minutes of the Fourteenth Annual Meeting of the American Medical Association; Report of the Committee of Publication; Report of the Treasurer; Address of Wilson Jewell, acting President of the Association; Report of the Committee on Medical Education, by Christopher C. Cox, Surgeon U. S. Volunteers, of Maryland; Report of the Committee on

Medical Literature, by Charles A. Lee, M. D.; Diatheses—their Surgical Relations and Effects, by E, Andrews, A. M., M. D., Prof. of Surgery in the Medical Department of Lind University; The American Method of Treating Joint Diseases and Deformities, by Henry G. Davis, New York; Cases of Diarrhœa Adiposa, by John H. Griscom, M. D., Physician of the New York Hospital; Report on American Necrology, by Christopher C. Cox, Surgeon United States Volunteers; An Inquiry into the Physiological and Medicinal Properties of the Veratrum Viride, together with some Physiological and Chemical Observations upon the Alkaloid Veratria obtained from this and other species: being the Prize Essay to which the American Medical Association awarded the Gold Medal for 1863, by Samuel R. Percy, M. D., Professor of Materia Medica and Therapeutics in the New Nork Medical College; Laryngoscopal Therapy, or the Medication of the Larynx under Sight, by Louis Elsberg, A. M., M. D., Lecturer on Diseases of the Throat in the University of New York, Fellow of the New York Academy of Medicine, Delegate from New York Med. Chir. College; Plan of Organization of the American Medical Association; Code of Ethics of the American Medical Association; Officers and Permanent Members.

